# Clinical Characteristics, Maternal and Neonatal Outcomes in Women with Placenta Previa Compared with Breech Cesarean Controls: A Retrospective Case-Control Study from a Single Tertiary Center in Lithuania

**DOI:** 10.3390/medicina62050931

**Published:** 2026-05-10

**Authors:** Vytaute Rimdzeviciute, Marija Leipuviene, Egle Savukyne, Laima Maleckiene, Gitana Ramoniene, Kotryna Bajeruniene, Mindaugas Kliucinskas

**Affiliations:** 1 Department of Obstetrics and Gynecology, Hospital of Lithuanian University of Health Sciences Kauno Klinikos, LT-50161 Kaunas, Lithuania; 2 Department of Obstetrics and Gynecology, Faculty of Medicine, Lithuanian University of Health Sciences, LT-44307 Kaunas, Lithuania; marija.leipuviene@lsmu.lt (M.L.); egle.savukyne@lsmu.lt (E.S.); laima.maleckiene@lsmu.lt (L.M.); gitana.ramoniene@lsmu.lt (G.R.); mindaugas.kliucinskas@lsmu.lt (M.K.); 3 Department of Obstetrics and Gynecology, Republican Panevezys Hospital, LT-35144 Panevezys, Lithuania; kotryna.bajeruniene@gmail.com

**Keywords:** placenta previa, placenta accreta, risk factors, outcomes

## Abstract

*Background and Objectives*: To evaluate maternal characteristics associated with placenta previa in comparison with breech cesarean controls, as well as maternal and neonatal outcomes. *Materials and Methods*: A retrospective case–control study was conducted at the Hospital of Lithuanian University of Health Sciences, Kaunas Clinics (2015–2022). A total of 150 cases of placenta previa were compared with a control group of participants who underwent cesarean delivery due to fetal breech presentation without placenta previa. *Results*: In multivariable analysis, higher parity, prior cesarean delivery, in vitro fertilization, prior surgical termination of pregnancy, and prior uterine surgery were independently associated with placenta previa compared with breech cesarean controls. Maternal complications were significantly more frequent in the placenta previa group and included placenta accreta spectrum disorders, second- and third-trimester hemorrhage, postpartum hemorrhage, and increased need for blood transfusion. The most severe outcomes, including cesarean hysterectomy, occurred exclusively in cases complicated by placenta accreta spectrum disorders. Neonatal outcomes in the placenta previa group were characterized by higher rates of preterm birth, low Apgar scores, and birth weight < 2500 g. Adverse neonatal outcomes were partly associated with earlier gestational age at delivery. However, placenta previa remained associated with low Apgar score after adjustment. *Conclusions*: Compared with breech cesarean controls, placenta previa was associated with multiple maternal characteristics, including higher parity, prior cesarean delivery, in vitro fertilization, prior surgical termination of pregnancy, and prior uterine surgery. The condition is linked to increased maternal hemorrhagic morbidity, particularly in cases complicated by placenta accreta spectrum disorders, as well as adverse neonatal outcomes mainly related to prematurity. These findings highlight the importance of careful antenatal monitoring and delivery planning in specialized centers.

## 1. Introduction

Placenta previa (PP) is a condition in which the placenta is implanted in the lower uterine segment, completely or partially covering the internal cervical os [1]. Although PP rates differ among studies, it occurs in approximately 0.3–1.3% of all pregnancies [2,3,4,5,6,7]. Risk factors associated with PP development have been extensively reviewed in the literature. Advanced maternal age, parity, prior cesarean section, infertility treatment, use of assisted reproductive technologies, and smoking are consistently identified as significant risk factors for PP [2,3,4,5,6,8,9,10,11]. A recent umbrella review further highlighted prior induced and spontaneous abortions, male fetal sex as highly suggestive risk factors [9]. In contrast, endometriosis and maternal cocaine use were classified as risk factors with limited supporting evidence [9]. PP is associated with increased maternal and neonatal morbidity and mortality. Maternal complications are mainly related to antepartum and postpartum hemorrhage, which significantly increase the risk of adverse outcomes [12,13]. Pregnancies complicated by PP are particularly prone to bleeding during the second or third trimester, increasing the risk of maternal and perinatal complications compared to the general population [14]. Additionally, PP is often complicated by placenta accreta spectrum (PAS) disorders [15], which can lead to severe postpartum hemorrhage. These disorders are associated with complications and adverse outcomes, including postpartum hysterectomy [5,7]. Neonatal morbidity and mortality are linked to preterm birth and its complications, including the need for neonatal intensive care unit (NICU) admission [16,17,18]. Some studies have reported a slight increase in the overall incidence of congenital anomalies in pregnancies complicated by PP. However, no specific anomaly or syndrome has been associated with the condition [2,19]. The relationship between PP and fetal growth restriction remains controversial [2,19]. Despite extensive research across various countries evaluating maternal characteristics, complications, and pregnancy outcomes associated with PP, no such study has been conducted in Lithuania.

## 2. Materials and Methods

**Figure 1:**
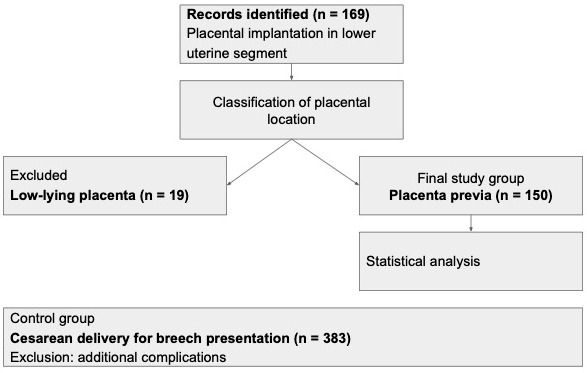
Flowchart of study population selection.

A retrospective case–control study was conducted to analyze cases of PP between 2015 and 2022. The study data from the Department of Obstetrics and Gynecology at the Hospital of the Lithuanian University of Health Sciences Kaunas Clinics, a tertiary care university hospital and one of Lithuania’s two referral centers for high-risk obstetric patients. Initially, 169 patients with singleton pregnancies and a prenatal diagnosis of placental implantation in the lower uterine segment were identified using the search term “placenta previa” from electronic medical records and hospital databases. These cases were further classified based on placental location. Nineteen cases were identified as low-lying placenta (defined as placental edge within 20 mm of the internal cervical os without covering it) and were excluded from the main comparative analysis due to their different clinical characteristics and outcome profile. The final study group consisted of 150 patients with placenta previa, defined as placental tissue partially or completely covering the internal cervical os. To avoid complications arising from other pathologies, the control group consisted of patients who underwent cesarean section (CS) due to breech presentation without PP. Cases in which the decision to perform CS in breech presentation was due to other indications, such as preeclampsia, premature rupture of membranes, or chorioamnionitis, were excluded from the study. The selection process of the study population is presented in Figure 1. The following clinical characteristics were evaluated: maternal age, parity, gestational age, and birth weight. Obstetric characteristics assessed included previous cesarean deliveries, pregnancies after assisted reproductive technology (ART), prior surgical termination of pregnancy, and uterine surgeries. Maternal complications and outcomes assessed included bleeding in the second and third trimesters, PAS, postpartum hemorrhage (quantitatively defined as cumulative blood loss ≥ 1000 mL during or following cesarean delivery), maternal blood transfusion (with indications based on both postpartum hemoglobin levels and clinical assessment), and the use of internal iliac artery balloon occlusion. The decision to perform balloon occlusion was individualized based on multidisciplinary assessment (suspected PAS, imaging findings, and hemorrhage risk), as no standardized institutional protocol was in place and availability of interventional radiology varied over time. Neonatal complications and outcomes assessed included prematurity, Apgar scores at 1 and 5 min, intrauterine growth restriction, and perinatal death.

Statistical analysis was performed using IBM SPSS Statistics version 29. Quantitative variables that did not meet the criteria for normality were described using medians and ranges (minimum–maximum). Differences between groups were compared using the Mann–Whitney U test for independent samples. Statistical significance for differences in qualitative variables was assessed using the Chi-square test or Fisher’s exact test, as appropriate. A multivariable logistic regression analysis was performed to identify factors independently associated with placenta previa compared with breech cesarean controls. Variables were selected based on clinical relevance and univariate differences between the PP and breech cesarean control groups. Adjusted odds ratios (aOR) with 95% confidence intervals were calculated. A backward stepwise likelihood ratio procedure was used, and variables retained in the final model were reported as independent factors. In addition, multivariable logistic regression analyses were performed for selected neonatal outcomes, adjusting for gestational age to account for its potential confounding effect. A *p*-value < 0.05 was considered statistically significant. Ethical approval was obtained from the Kaunas Regional Ethics Committee (protocol No. 2026-BE10-0002 4 March 2026).

## 3. Results

Placenta previa (PP) complicated 150 pregnancies during the study period, defined as placental tissue partially or completely covering the internal cervical os. The median maternal age in the PP group was 33 years (range 19–46), compared with 30 years (range 16–43) in the control group (*p* < 0.001). Women in the PP group had a higher median parity (2; range 1–9) than those in the control group (1; range 1–6) (*p* < 0.001). In multivariable logistic regression analysis, parity, previous cesarean delivery, in vitro fertilization (IVF), prior surgical termination of pregnancy, and prior uterine surgery were independently associated with PP compared with breech cesarean controls (see Table 1). IVF showed the strongest association (adjusted odds ratio [aOR] 6.70, 95% confidence interval [CI] 2.73–16.43). Previous uterine surgery (aOR 5.86, 95% CI 2.84–12.10) and prior surgical termination of pregnancy (aOR 3.53, 95% CI 1.64–7.60) were also strongly associated with PP. Prior cesarean delivery increased the risk approximately 2.5-fold (aOR 2.49, 95% CI 1.46–4.25), while higher parity was likewise associated with increased risk (aOR 2.17, 95% CI 1.62–2.91). Among women with PP, 36 (24.0%) had one previous cesarean delivery, 16 (10.7%) had two, 4 (2.7%) had three, and 2 (1.3%) had four prior cesarean deliveries.

PP was significantly associated with maternal complications and adverse outcomes. Specifically, PP was linked to PAS disorders, hemorrhage in the second and third trimesters, postpartum hemorrhage, maternal blood transfusions, and cesarean hysterectomy. Postpartum hemorrhage was defined as cumulative blood loss ≥ 1000 mL during or following cesarean delivery. The incidence of postpartum hemorrhage was significantly higher in the PP group (*p* < 0.001), with a median blood loss of 1500 mL (range 1000–5000 mL). A detailed list of maternal complications and outcomes is presented in Table 2.

No significant differences were observed in the rates of emergency CS between the PP and control groups (58 (38.7%) vs. 144 (37.6%), *p* = 0.447). Antepartum bleeding was one of the indications to perform emergency cesarean section and occurred in 30 PP cases (30%), with a median blood loss of 400 mL (range 100–1700 mL). The rates of postpartum hemorrhage did not differ between emergency and planned CSs (27 (46.6%) vs. 37 (40.2%), *p* = 0.445). In 12 cases (8.0%) of PP, internal iliac artery balloon occlusion was used to reduce postpartum hemorrhage. However, there was no significant difference in hemorrhage rates between patients who underwent internal iliac artery balloon occlusion and those who did not (*p* = 0.496). The median blood loss was 1450 mL (range 1100–1500 mL) in the internal iliac artery balloon occlusion group and 1500 mL (range 1000–5000 mL) in the non-occlusion group (*p* = 0.086).

Maternal complications and adverse outcomes in the PP group were more frequent in cases complicated by PAS disorders, which affected 40 cases (26.7%) within this group. Among these, 15 cases (10.0%) involved placenta accreta, 18 cases (12.0%) involved placenta increta, and 7 cases (4.7%) involved placenta percreta. In PAS cases, placental attachment to the cesarean scar was significantly more common compared to PP without PAS (19 (47.5%) vs. 14 (12.7%), OR 6.204, 95% CI 2.688–14.319, *p* < 0.001). The incidence of postpartum hemorrhage was significantly higher in PAS cases (*p* < 0.001). In PAS cases, the median blood loss was 1900 mL (range 1200–5000 mL), compared to 1500 mL (range 1000–2500 mL) in PP cases without PAS (*p* < 0.001). All cesarean hysterectomies (n = 11) in the PP group occurred in patients with PAS, including 6 cases (54.5%) of placenta percreta and 5 cases (45.5%) of placenta increta (see Table 3). Although cesarean hysterectomy was exclusively associated with PAS, hemorrhage-related complications were still more frequent in PP without PAS compared to the control group (see Table 4). However, no significant difference in median blood loss was observed between these groups (1500 mL (range 1000–2500 mL) vs. 1500 mL (range 1100–2000 mL), respectively, *p* = 0.425). Importantly, even after excluding PAS cases, PP remained associated with increased hemorrhagic morbidity compared with the control group.

Neonatal complications included preterm birth, low Apgar scores at 1 and 5 min, and birth weight < 2500 g. No significant differences in the rates of intrauterine growth restriction were observed (see Table 5). Two cases (1.2%) of postnatal death were reported, both associated with prematurity at 25 and 26 weeks of gestation. In addition, differences in birth weight were observed, with a median of 3085 g (range 544–4275) in the PP group compared to 3185 g (range 1076–4690) in the control group (*p* < 0.001). The median gestational age at delivery in the PP group was significantly lower compared to the control group, with values of 37 weeks (range 24–41) and 39 weeks (range 28–41), respectively (*p* < 0.001). Significant differences were also noted in the gestational age at delivery between planned and emergency cesarean sections within the PP group, with medians of 37 weeks (range 33–40) and 35 weeks (range 24–41), respectively (*p* < 0.001).

In multivariable logistic regression analysis adjusted for gestational age, placenta previa remained significantly associated with low Apgar score at 1 min (aOR 3.38, 95% CI 1.47–7.77, *p* = 0.004), while gestational age was also independently associated with this outcome (aOR 6.16, 95% CI 2.64–14.37, *p* < 0.001).

In contrast, after adjustment for gestational age, the association between placenta previa and birth weight < 2500 g was no longer significant (aOR 0.76, 95% CI 0.40–1.43, *p* = 0.388), suggesting that this relationship is largely explained by earlier gestational age at delivery.

## 4. Discussion

This retrospective study presents an analysis of pregnancies complicated by PP and highlights associated maternal characteristics, as well as maternal and neonatal complications and outcomes. Our findings are consistent with the existing literature and provide additional insights into this pathology.

### 4.1. Maternal Characteristics Associated with PP

In this study, several maternal characteristics previously reported in association with PP were more frequent among women with PP compared with breech cesarean controls. In our study maternal age was significantly higher in the placenta previa group in univariate analysis; however, it was not independently associated with placenta previa in the multivariable model. Advanced maternal age has long been recognized as a risk factor for PP, possibly due to age-related changes in endometrial vascularity and uterine receptivity that may lead to abnormal placental implantation [2,9]. Although advanced maternal age has been recognized as a risk factor for PP in previous studies, in our cohort it was significant only in univariate analysis and did not remain independently associated after adjustment. In multivariable analysis, higher parity, prior cesarean delivery, IVF, prior surgical termination of pregnancy, and prior uterine surgery remained independently associated with PP. Higher parity was significantly associated with PP in our cohort. This finding supports previous studies suggesting that higher parity may increase the risk of placental implantation in the lower uterine segment [10]. Prior cesarean delivery was significantly associated with PP compared with breech cesarean controls. Previous uterine surgery, including cesarean delivery and surgical abortion, has been consistently associated with PP and PAS disorders due to scar tissue formation and altered endometrial repair mechanisms [3,4]. In addition, a history of PP or PAS disorders was observed only in the PP group, which is consistent with previous reports suggesting an increased risk of recurrence. IVF showed the strongest association with PP in our study and remained independently associated in the multivariable model. This finding is consistent with accumulating evidence suggesting that ART, particularly IVF, may be associated with abnormal placentation [1]. Proposed mechanisms include altered implantation timing and hormonal influences on endometrial receptivity during embryo transfer cycles. Interestingly, smoking and hypertensive disorders were not significantly associated with PP in univariate analysis. While smoking has been implicated in the etiology of placenta previa in some studies [20], its role remains controversial. In our study, this finding may be influenced by underreporting or incomplete documentation of smoking status, a limitation also noted in other retrospective analyses [5]. A recent umbrella review also highlighted spontaneous and induced abortions, as well as male fetal sex, as potential risk factors for placenta previa [9]. In the present study, only prior surgical termination of pregnancy was evaluated, while spontaneous and medically induced abortions were not included in the analysis. Overall, this study highlights several maternal and obstetric characteristics associated with PP in this specific comparison group. Although multivariable analysis was performed, the observational nature of the study limits causal interpretation, and the identified factors should be considered as associations rather than causes.

### 4.2. Maternal Complications in Pregnancies Complicated by PP

In our study, the PP group demonstrated a higher incidence of hemorrhage, particularly in the second and third trimesters and during the postpartum period, compared with the selected control group, consistent with trends reported in earlier literature [12,13].

Antepartum bleeding is likely related to the impaired placental attachment in the lower uterine segment. This region undergoes significant stretching and thinning in the third trimester, which may lead to premature placental detachment and bleeding [14]. The occurrence of bleeding in late pregnancy not only affects maternal hemodynamic stability but also increases the likelihood of emergency delivery and prematurity-related neonatal complications. One of the most critical complications associated with PP is the presence of PAS disorders. These disorders were strongly associated with postpartum hemorrhage, increased blood loss, and the need for cesarean hysterectomy. In addition, patients with PAS more frequently required blood transfusion. All hysterectomy cases occurred in patients with PAS, showing the severity of these disorders and their impact on maternal outcomes. The literature reports varying PAS incidence rates in women with PP, ranging from 2.9% to as high as 71.6%, depending on population characteristics and diagnostic criteria [5]. In our cohort, the observed rate is within this range and highlights the need for careful prenatal diagnosis, particularly in women with known risk factors. Although internal iliac artery balloon occlusion was used in some PP cases to reduce intraoperative blood loss, no statistically significant benefit was observed in our cohort. This may be explained by differences in technique, timing, and case selection [21]. More standardized protocols and prospective evaluations are necessary to determine the precise role of prophylactic vascular interventions in managing high-risk PP cases.

Importantly, our analysis demonstrated that a substantial proportion of severe maternal morbidity was attributable to PAS disorders. However, even in the absence of PAS, placenta previa itself remained associated with increased hemorrhagic risk compared to the control group, suggesting that both conditions independently contribute to adverse outcomes.

### 4.3. Neonatal Complications in Pregnancies Complicated by PP

Neonatal morbidity and mortality in pregnancies complicated by PP are closely related to the higher rates of preterm birth and its associated complications. Consistent with previous studies, our findings demonstrated a significantly lower median gestational age in the PP group compared to the control group, with an even earlier gestational age observed among women undergoing emergency cesarean sections. These results align with prior literature identifying PP as a strong predictor of medically indicated or spontaneous preterm delivery, which subsequently increases the risk of neonatal complications [16,17,18]. In our study, neonatal complications in the PP group included lower Apgar scores at 1 and 5 min and a higher proportion of infants with birth weight < 2500 g. In multivariable analysis adjusted for gestational age, placenta previa remained significantly associated with low Apgar score at 1 min, whereas the association with low birth weight was no longer significant, indicating that this relationship is largely explained by prematurity. Overall, these findings suggest that adverse neonatal outcomes are partly mediated by earlier gestational age at delivery; however, the persistence of low Apgar scores after adjustment indicates that additional factors beyond gestational age may contribute. This finding supports previous reports suggesting an inconsistent relationship between PP and IUGR, with some studies indicating no significant impact on fetal growth, while others suggest possible placental dysfunction due to abnormal implantation [2,19]. Recent studies have also explored early prediction of preterm birth using first-trimester biochemical markers, further supporting the central role of prematurity in adverse neonatal outcomes [22]. Furthermore, recent studies indicate that combined first-trimester screening parameters, including maternal characteristics, biochemical markers, and ultrasound findings, may identify pregnancies at increased risk of neonatal complications; however, their predictive performance remains limited, reflecting the multifactorial nature of these outcomes [23]. In this context, placenta previa may represent one component of a broader high-risk obstetric profile, in which neonatal morbidity is largely mediated by prematurity rather than placental location itself. Two cases of neonatal death were recorded, both attributed to extreme prematurity. Although rare, these outcomes underscore the critical importance of gestational age in determining neonatal survival. Our data highlight that earlier gestational age at delivery—particularly in the context of emergency cesarean section—remains one of the most influential risk factors for neonatal morbidity and mortality in PP pregnancies.

### 4.4. Strengths and Limitations

The main strength of this study is the relatively large sample size and detailed data collection, which allowed us to evaluate maternal and obstetric characteristics as well as maternal and neonatal outcomes associated with PP. However, the study has several limitations. As this was a retrospective single-center study, our findings may be limited. Moreover, the study did not assess long-term neonatal outcomes, which limits the understanding of extended impacts of PP-associated complications on child development. An additional limitation of this study is the selection of the control group, which consisted of women undergoing cesarean delivery due to breech presentation rather than a general obstetric population. This may introduce selection bias and limits the interpretation of the identified associations as causal risk factors.

### 4.5. Implications for Future Research

Future studies using population-based or general obstetric control groups should focus on developing standardized models to identify women at increased likelihood of PP and PAS. More studies are also needed to better understand long-term outcomes in children born to mothers with PP. Furthermore, randomized studies are needed to evaluate different strategies for hemorrhage control. A better understanding of the underlying mechanisms of PP and PAS may also improve early diagnosis and management.

## 5. Conclusions

PP remains an important obstetric condition associated with increased maternal and neonatal morbidity. In this study, advanced maternal age was associated with PP only in univariate analysis and did not remain independently associated after adjustment. Compared with breech cesarean controls, PP was independently associated with higher parity, prior uterine surgery, prior cesarean delivery, prior surgical termination of pregnancy, and in vitro fertilization (IVF). Recognition of these associated characteristics may help inform antenatal assessment and monitoring, although the findings should be interpreted in the context of the selected breech cesarean control group. Maternal complications were primarily related to bleeding, including antepartum and postpartum hemorrhage, with the most severe outcomes, such as blood transfusion and cesarean hysterectomy, occurring in cases complicated by PAS disorders. Neonatal outcomes were partly affected by prematurity, with lower birth weights and Apgar scores more frequently observed in the PP group, particularly in emergency deliveries. However, placenta previa remained associated with low Apgar scores after adjustment for gestational age. Overall, pregnancies complicated by PP require careful antenatal follow-up, timely diagnosis, and well-coordinated delivery planning.

## Figures and Tables

**Table 1:** Multivariable logistic regression analysis of factors associated with placenta previa compared with breech cesarean controls.

Variable	aOR (95% CI)	*p* Value
Parity (per 1 increase)	2.17 (1.62–2.91)	<0.001
Previous cesarean delivery	2.49 (1.46–4.25)	<0.001
In vitro fertilization (IVF)	6.70 (2.73–16.43)	<0.001
Prior surgical termination of pregnancy	3.53 (1.64–7.60)	<0.001
Prior uterine surgery	5.86 (2.84–12.10)	<0.001

Notes: Data are presented as adjusted odds ratios (aOR) with 95% confidence intervals (CI). CI—confidence interval; IVF—in vitro fertilization. *p* values were calculated using multivariable logistic regression analysis. Variables included in the model were selected based on clinical relevance and univariate analysis.

**Table 2:** Maternal outcomes associated with placenta previa compared with the control group.

Maternal Outcome	Placenta Previa (*n* = 150), *n* (%)	Control Group (*n* = 383), *n* (%)	OR	95% CI	*p* Value
Placenta accreta spectrum disorders	40 (26.7)	8 (2.1)	12.76	6.12–26.63	<0.001
Hemorrhage in the second trimester of pregnancy	28 (18.7)	1 (0.3)	71.49	9.81–520.76	<0.001
Hemorrhage in the third trimester of pregnancy ^a^	82 (56.6)	4 (1.0)	54.14	20.21–145.03	<0.001
Postpartum hemorrhage ^b^	64 (42.7)	9 (2.3)	18.15	9.27–35.54	<0.001
Maternal blood transfusion	25 (16.7)	4 (1.0)	15.95	5.64–45.08	<0.001
Cesarean hysterectomy	11 (7.3)	0 (0.00)	not estimated	not estimated	<0.001

Notes: Data are presented as *n* (%). OR—odds ratio; CI—confidence interval. *p* values were calculated using the χ^2^ test or Fisher’s exact test, as appropriate. ^a^ Sample size differed because five patients delivered before the beginning of the third trimester (*n* = 145). ^b^ Postpartum hemorrhage was defined as cumulative blood loss ≥ 1000 mL during or following cesarean delivery.

**Table 3:** Complications in PAS group compared with placenta previa without PAS.

Maternal Complications	PAS (*n* = 40), *n* (%)	PP Without PAS (*n* = 110), *n* (%)	OR	95% CI	*p* Value
Postpartum hemorrhage	33 (82.5)	31 (28.2)	12.01	4.81–30.00	<0.001
Cesarean hysterectomy	11 (25.0)	0	not estimated	not estimated	<0.001
Maternal blood transfusion	17 (42.5)	8 (7.3)	9.42	3.62–24.47	<0.001

Notes: Data are presented as *n* (%). OR—odds ratio; CI—confidence interval. *p* values were calculated using the χ^2^ test or Fisher’s exact test, as appropriate.

**Table 4:** Complications in placenta previa without PAS compared with the control group.

Maternal Complications	PP Without PAS (*n* = 110), *n* (%)	Control Group Without PAS (*n* = 375), *n* (%)	OR	95% CI	*p* Value
Postpartum hemorrhage	31 (28.2)	9 (2.4)	11.74	5.76–23.90	<0.001
Maternal blood transfusion	8 (7.3)	4 (1.1)	6.81	2.09–22.21	<0.001

Notes: Data are presented as *n* (%). OR—odds ratio; CI—confidence interval. *p* values were calculated using the χ^2^ test or Fisher’s exact test, as appropriate.

**Table 5:** Neonatal outcomes associated with placenta previa compared with the control group.

Neonatal Outcome	Placenta Previa (*n* = 150), *n* (%)	Control Group (*n* = 383), *n* (%)	OR	95% CI	*p* Value
Preterm birth	62 (41.3)	62 (16.2)	2.55	1.89–3.43	<0.001
Apgar score at 1 min < 7	19 (14.2)	10 (2.6)	4.85	2.31–10.18	<0.001
Apgar score at 5 min < 7	6 (3.6)	0 (0.0)	not estimated	not estimated	0.002
Birth weight < 2500 g	37 (24.7)	51 (13.3)	1.85	1.26–2.70	0.002
Perinatal mortality	2 (1.3)	0 (0.0)	not estimated	not estimated	0.079
IUGR	14 (9.3)	30 (7.8)	1.19	0.65–2.18	0.571

Notes: Data are presented as *n* (%). OR—odds ratio; CI—confidence interval; IUGR—intrauterine growth restriction. These are unadjusted odds ratios. *p* values were calculated using the χ^2^ test or Fisher’s exact test, as appropriate.

## Data Availability

The data presented in this study are available on request from the corresponding author due to ethical and privacy restrictions related to patient confidentiality.

## Abbreviations

PP	Placenta previa
PAS	Placenta accreta spectrum
NICU	Neonatal intensive care unit
CS	Cesarean section
ART	Assisted reproductive technology
OR	Odds ratio
